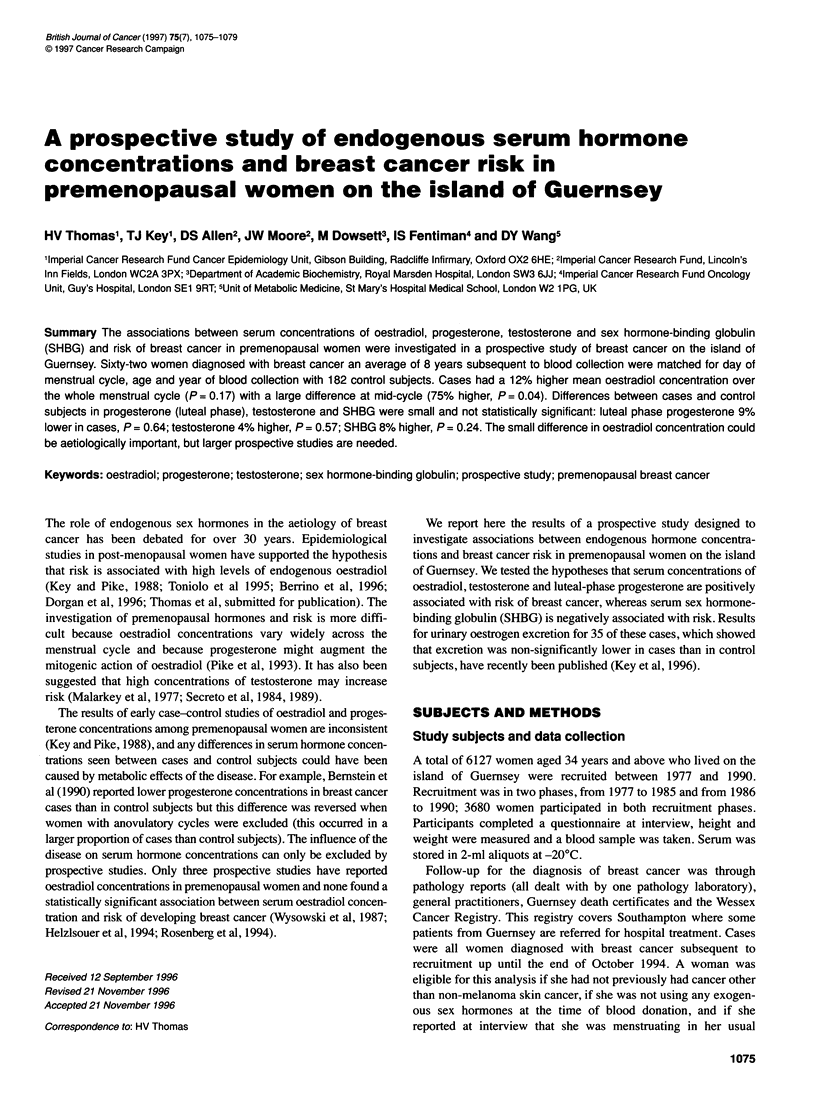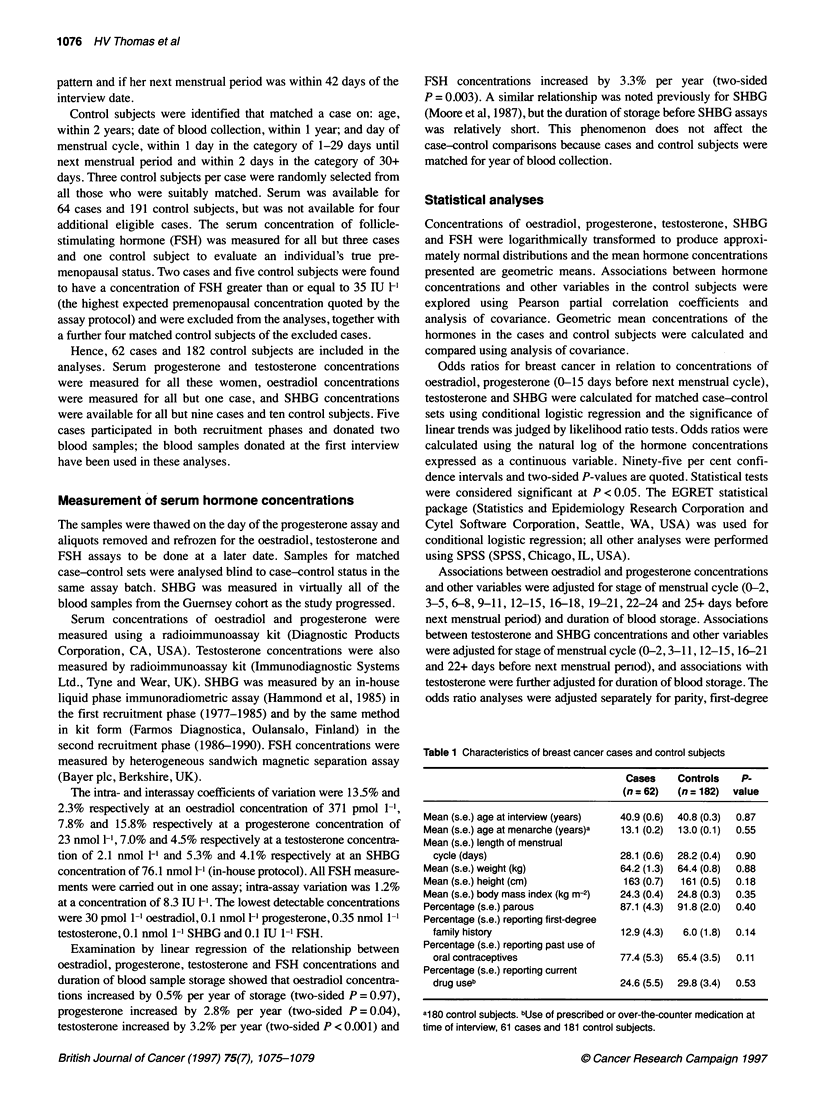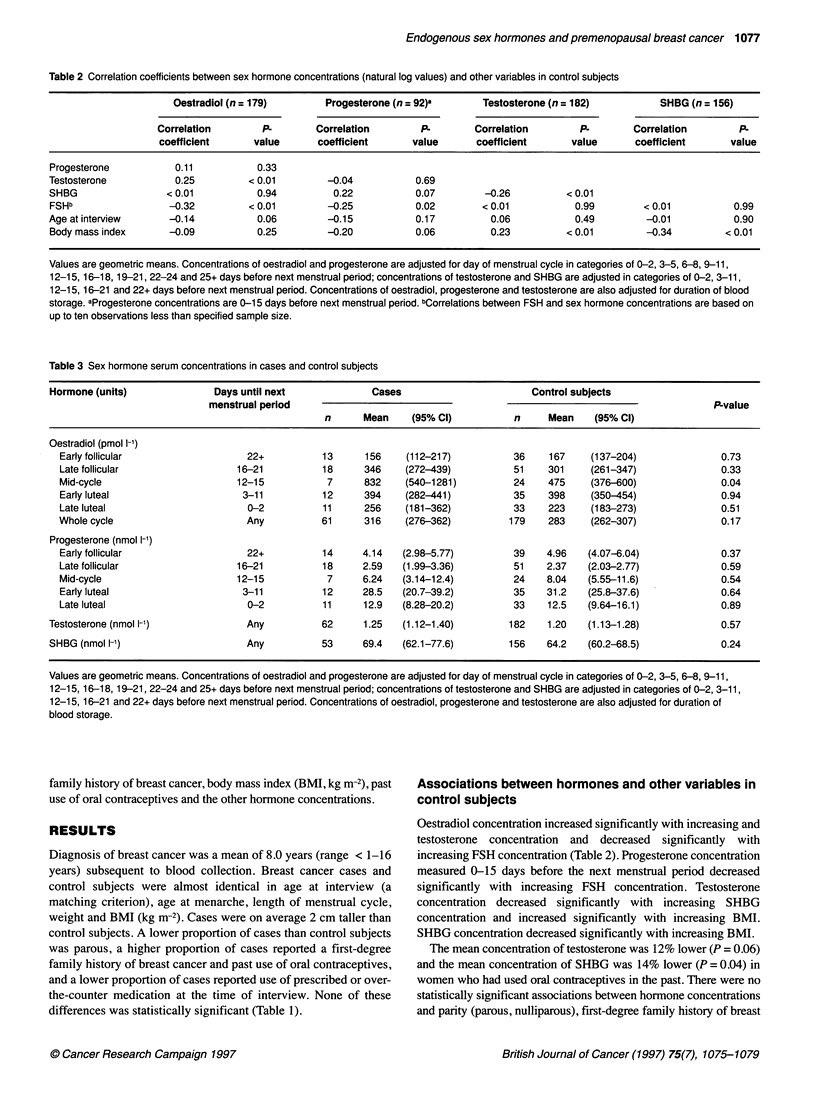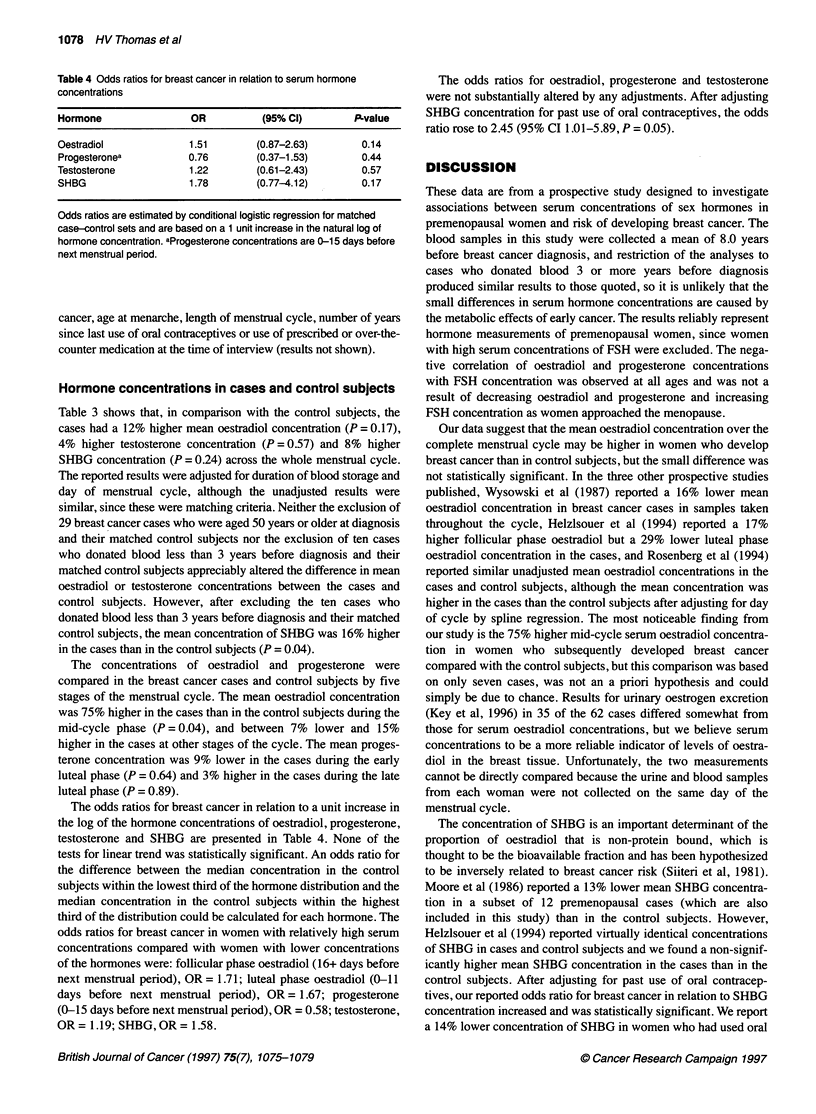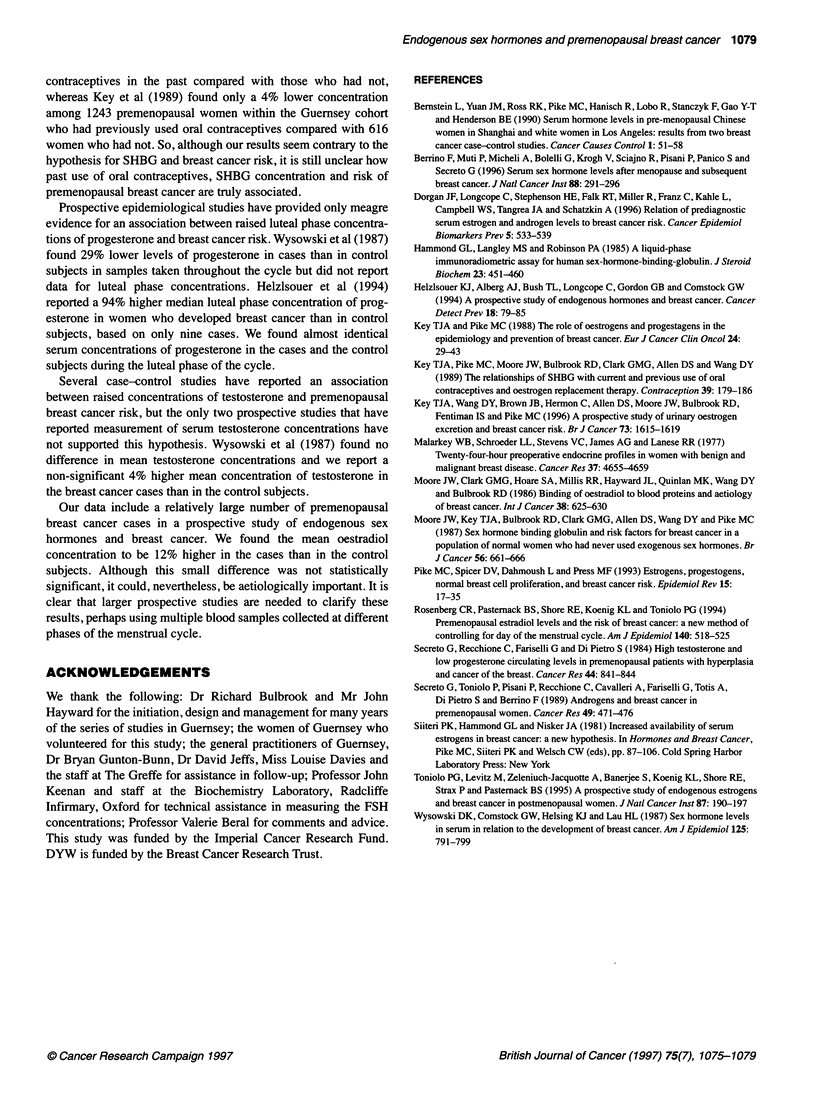# A prospective study of endogenous serum hormone concentrations and breast cancer risk in premenopausal women on the island of Guernsey.

**DOI:** 10.1038/bjc.1997.183

**Published:** 1997

**Authors:** H. V. Thomas, T. J. Key, D. S. Allen, J. W. Moore, M. Dowsett, I. S. Fentiman, D. Y. Wang

**Affiliations:** Imperial Cancer Research Fund Cancer Epidemiology Unit, Radcliffe Infirmary, Oxford, UK.

## Abstract

The associations between serum concentrations of oestradiol, progesterone, testosterone and sex hormone-binding globulin (SHBG) and risk of breast cancer in premenopausal women were investigated in a prospective study of breast cancer on the island of Guernsey. Sixty-two women diagnosed with breast cancer an average of 8 years subsequent to blood collection were matched for day of menstrual cycle, age and year of blood collection with 182 control subjects. Cases had a 12% higher mean oestradiol concentration over the whole menstrual cycle (P = 0.17) with a large difference at mid-cycle (75% higher, P = 0.04). Differences between cases and control subjects in progesterone (luteal phase), testosterone and SHBG were small and not statistically significant: luteal phase progesterone 9% lower in cases, P = 0.64; testosterone 4% higher, P = 0.57; SHBG 8% higher, P = 0.24. The small difference in oestradiol concentration could be aetiologically important, but larger prospective studies are needed.


British Journal of Cancer (1997) 75(7), 1075–1079
© 1997 Cancer Research Campaign

### A prospective study of endogenous serum hormone concentrations and breast cancer risk in premenopausal women on the island of Guernsey

**HV Thomas[1], TJ Key[1], DS Allen[2], JW Moore[2], M Dowsett[3], IS Fentiman[4] and DY Wang[5]**

[1]Imperial Cancer Research Fund Cancer Epidemiology Unit, Gibson Building, Radcliffe Infirmary, Oxford OX2 6HE; [2]Imperial Cancer Research Fund, Lincoln's Inn Fields, London WC2A 3PX; [3]Department of Academic Biochemistry, Royal Marsden Hospital, London SW3 6JJ; [4]Imperial Cancer Research Fund Oncology Unit, Guy's Hospital, London SE1 9RT; [5]Unit of Metabolic Medicine, St Mary's Hospital Medical School, London W2 1PG, UK

**Summary** The associations between serum concentrations of oestradiol, progesterone, testosterone and sex hormone-binding globulin (SHBG) and risk of breast cancer in premenopausal women were investigated in a prospective study of breast cancer on the island of Guernsey. Sixty-two women diagnosed with breast cancer an average of 8 years subsequent to blood collection were matched for day of menstrual cycle, age and year of blood collection with 182 control subjects. Cases had a 12% higher mean oestradiol concentration over the whole menstrual cycle ($P = 0.17$) with a large difference at mid-cycle (75% higher, $P = 0.04$). Differences between cases and control subjects in progesterone (luteal phase), testosterone and SHBG were small and not statistically significant: luteal phase progesterone 9% lower in cases, $P = 0.64$; testosterone 4% higher, $P = 0.57$; SHBG 8% higher, $P = 0.24$. The small difference in oestradiol concentration could be aetiologically important, but larger prospective studies are needed.

**Keywords:** oestradiol; progesterone; testosterone; sex hormone-binding globulin; prospective study; premenopausal breast cancer

The role of endogenous sex hormones in the aetiology of breast cancer has been debated for over 30 years. Epidemiological studies in post-menopausal women have supported the hypothesis that risk is associated with high levels of endogenous oestradiol (Key and Pike, 1988; Toniolo et al 1995; Berrino et al, 1996; Dorgan et al, 1996; Thomas et al, submitted for publication). The investigation of premenopausal hormones and risk is more difficult because oestradiol concentrations vary widely across the menstrual cycle and because progesterone might augment the mitogenic action of oestradiol (Pike et al, 1993). It has also been suggested that high concentrations of testosterone may increase risk (Malarkey et al, 1977; Secreto et al, 1984, 1989).

The results of early case–control studies of oestradiol and progesterone concentrations among premenopausal women are inconsistent (Key and Pike, 1988), and any differences in serum hormone concentrations seen between cases and control subjects could have been caused by metabolic effects of the disease. For example, Bernstein et al (1990) reported lower progesterone concentrations in breast cancer cases than in control subjects but this difference was reversed when women with anovulatory cycles were excluded (this occurred in a larger proportion of cases than control subjects). The influence of the disease on serum hormone concentrations can only be excluded by prospective studies. Only three prospective studies have reported oestradiol concentrations in premenopausal women and none found a statistically significant association between serum oestradiol concentration and risk of developing breast cancer (Wysowski et al, 1987; Helzlsouer et al, 1994; Rosenberg et al, 1994).

*Received 12 September 1996*
*Revised 21 November 1996*
*Accepted 21 November 1996*

*Correspondence to*: HV Thomas

We report here the results of a prospective study designed to investigate associations between endogenous hormone concentrations and breast cancer risk in premenopausal women on the island of Guernsey. We tested the hypotheses that serum concentrations of oestradiol, testosterone and luteal-phase progesterone are positively associated with risk of breast cancer, whereas serum sex hormone-binding globulin (SHBG) is negatively associated with risk. Results for urinary oestrogen excretion for 35 of these cases, which showed that excretion was non-significantly lower in cases than in control subjects, have recently been published (Key et al, 1996).

### SUBJECTS AND METHODS

### Study subjects and data collection

A total of 6127 women aged 34 years and above who lived on the island of Guernsey were recruited between 1977 and 1990. Recruitment was in two phases, from 1977 to 1985 and from 1986 to 1990; 3680 women participated in both recruitment phases. Participants completed a questionnaire at interview, height and weight were measured and a blood sample was taken. Serum was stored in 2-ml aliquots at –20°C.

Follow-up for the diagnosis of breast cancer was through pathology reports (all dealt with by one pathology laboratory), general practitioners, Guernsey death certificates and the Wessex Cancer Registry. This registry covers Southampton where some patients from Guernsey are referred for hospital treatment. Cases were all women diagnosed with breast cancer subsequent to recruitment up until the end of October 1994. A woman was eligible for this analysis if she had not previously had cancer other than non-melanoma skin cancer, if she was not using any exogenous sex hormones at the time of blood donation, and if she reported at interview that she was menstruating in her usual pattern and if her next menstrual period was within 42 days of the interview date.

Control subjects were identified that matched a case on: age, within 2 years; date of blood collection, within 1 year; and day of menstrual cycle, within 1 day in the category of 1–29 days until next menstrual period and within 2 days in the category of 30+ days. Three control subjects per case were randomly selected from all those who were suitably matched. Serum was available for 64 cases and 191 control subjects, but was not available for four additional eligible cases. The serum concentration of follicle-stimulating hormone (FSH) was measured for all but three cases and one control subject to evaluate an individual's true pre-menopausal status. Two cases and five control subjects were found to have a concentration of FSH greater than or equal to 35 IU $l^{-1}$ (the highest expected premenopausal concentration quoted by the assay protocol) and were excluded from the analyses, together with a further four matched control subjects of the excluded cases.

Hence, 62 cases and 182 control subjects are included in the analyses. Serum progesterone and testosterone concentrations were measured for all these women, oestradiol concentrations were measured for all but one case, and SHBG concentrations were available for all but nine cases and ten control subjects. Five cases participated in both recruitment phases and donated two blood samples; the blood samples donated at the first interview have been used in these analyses.

### Measurement of serum hormone concentrations

The samples were thawed on the day of the progesterone assay and aliquots removed and refrozen for the oestradiol, testosterone and FSH assays to be done at a later date. Samples for matched case–control sets were analysed blind to case–control status in the same assay batch. SHBG was measured in virtually all of the blood samples from the Guernsey cohort as the study progressed.

Serum concentrations of oestradiol and progesterone were measured using a radioimmunoassay kit (Diagnostic Products Corporation, CA, USA). Testosterone concentrations were also measured by radioimmunoassay kit (Immunodiagnostic Systems Ltd., Tyne and Wear, UK). SHBG was measured by an in-house liquid phase immunoradiometric assay (Hammond et al, 1985) in the first recruitment phase (1977–1985) and by the same method in kit form (Farmos Diagnostica, Oulansalo, Finland) in the second recruitment phase (1986–1990). FSH concentrations were measured by heterogeneous sandwich magnetic separation assay (Bayer plc, Berkshire, UK).

The intra- and interassay coefficients of variation were 13.5% and 2.3% respectively at an oestradiol concentration of 371 pmol $l^{-1}$, 7.8% and 15.8% respectively at a progesterone concentration of 23 nmol $l^{-1}$, 7.0% and 4.5% respectively at a testosterone concentration of 2.1 nmol $l^{-1}$ and 5.3% and 4.1% respectively at an SHBG concentration of 76.1 nmol $l^{-1}$ (in-house protocol). All FSH measurements were carried out in one assay; intra-assay variation was 1.2% at a concentration of 8.3 IU $l^{-1}$. The lowest detectable concentrations were 30 pmol $l^{-1}$ oestradiol, 0.1 nmol $l^{-1}$ progesterone, 0.35 nmol $l^{-1}$ testosterone, 0.1 nmol $l^{-1}$ SHBG and 0.1 IU $l^{-1}$ FSH.

Examination by linear regression of the relationship between oestradiol, progesterone, testosterone and FSH concentrations and duration of blood sample storage showed that oestradiol concentrations increased by 0.5% per year of storage (two-sided $P = 0.97$), progesterone increased by 2.8% per year (two-sided $P = 0.04$), testosterone increased by 3.2% per year (two-sided $P < 0.001$) and FSH concentrations increased by 3.3% per year (two-sided $P = 0.003$). A similar relationship was noted previously for SHBG (Moore et al, 1987), but the duration of storage before SHBG assays was relatively short. This phenomenon does not affect the case–control comparisons because cases and control subjects were matched for year of blood collection.

### Statistical analyses

Concentrations of oestradiol, progesterone, testosterone, SHBG and FSH were logarithmically transformed to produce approximately normal distributions and the mean hormone concentrations presented are geometric means. Associations between hormone concentrations and other variables in the control subjects were explored using Pearson partial correlation coefficients and analysis of covariance. Geometric mean concentrations of the hormones in the cases and control subjects were calculated and compared using analysis of covariance.

Odds ratios for breast cancer in relation to concentrations of oestradiol, progesterone (0–15 days before next menstrual cycle), testosterone and SHBG were calculated for matched case–control sets using conditional logistic regression and the significance of linear trends was judged by likelihood ratio tests. Odds ratios were calculated using the natural log of the hormone concentrations expressed as a continuous variable. Ninety-five per cent confidence intervals and two-sided $P$-values are quoted. Statistical tests were considered significant at $P < 0.05$. The EGRET statistical package (Statistics and Epidemiology Research Corporation and Cytel Software Corporation, Seattle, WA, USA) was used for conditional logistic regression; all other analyses were performed using SPSS (SPSS, Chicago, IL, USA).

Associations between oestradiol and progesterone concentrations and other variables were adjusted for stage of menstrual cycle (0–2, 3–5, 6–8, 9–11, 12–15, 16–18, 19–21, 22–24 and 25+ days before next menstrual period) and duration of blood storage. Associations between testosterone and SHBG concentrations and other variables were adjusted for stage of menstrual cycle (0–2, 3–11, 12–15, 16–21 and 22+ days before next menstrual period), and associations with testosterone were further adjusted for duration of blood storage. The odds ratio analyses were adjusted separately for parity, first-degree

**Table 1** Characteristics of breast cancer cases and control subjects

| | Cases ($n$ = 62) | Controls ($n$ = 182) | P-value |
|---|---|---|---|
| Mean (s.e.) age at interview (years) | 40.9 (0.6) | 40.8 (0.3) | 0.87 |
| Mean (s.e.) age at menarche (years)[a] | 13.1 (0.2) | 13.0 (0.1) | 0.55 |
| Mean (s.e.) length of menstrual cycle (days) | 28.1 (0.6) | 28.2 (0.4) | 0.90 |
| Mean (s.e.) weight (kg) | 64.2 (1.3) | 64.4 (0.8) | 0.88 |
| Mean (s.e.) height (cm) | 163 (0.7) | 161 (0.5) | 0.18 |
| Mean (s.e.) body mass index (kg $m^{-2}$) | 24.3 (0.4) | 24.8 (0.3) | 0.35 |
| Percentage (s.e.) parous | 87.1 (4.3) | 91.8 (2.0) | 0.40 |
| Percentage (s.e.) reporting first-degree family history | 12.9 (4.3) | 6.0 (1.8) | 0.14 |
| Percentage (s.e.) reporting past use of oral contraceptives | 77.4 (5.3) | 65.4 (3.5) | 0.11 |
| Percentage (s.e.) reporting current drug use[b] | 24.6 (5.5) | 29.8 (3.4) | 0.53 |

[a]180 control subjects. [b]Use of prescribed or over-the-counter medication at time of interview, 61 cases and 181 control subjects.

© Cancer Research Campaign 1997

**Table 2** Correlation coefficients between sex hormone concentrations (natural log values) and other variables in control subjects

| | Oestradiol ($n$ = 179) | | Progesterone ($n$ = 92)[a] | | Testosterone ($n$ = 182) | | SHBG ($n$ = 156) | |
|---|---|---|---|---|---|---|---|---|
| | Correlation coefficient | P-value | Correlation coefficient | P-value | Correlation coefficient | P-value | Correlation coefficient | P-value |
| Progesterone | 0.11 | 0.33 | | | | | | |
| Testosterone | 0.25 | < 0.01 | –0.04 | 0.69 | | | | |
| SHBG | < 0.01 | 0.94 | 0.22 | 0.07 | –0.26 | < 0.01 | | |
| FSH[b] | –0.32 | < 0.01 | –0.25 | 0.02 | < 0.01 | 0.99 | < 0.01 | 0.99 |
| Age at interview | –0.14 | 0.06 | –0.15 | 0.17 | 0.06 | 0.49 | –0.01 | 0.90 |
| Body mass index | –0.09 | 0.25 | –0.20 | 0.06 | 0.23 | < 0.01 | –0.34 | < 0.01 |

Values are geometric means. Concentrations of oestradiol and progesterone are adjusted for day of menstrual cycle in categories of 0–2, 3–5, 6–8, 9–11, 12–15, 16–18, 19–21, 22–24 and 25+ days before next menstrual period; concentrations of testosterone and SHBG are adjusted in categories of 0–2, 3–11, 12–15, 16–21 and 22+ days before next menstrual period. Concentrations of oestradiol, progesterone and testosterone are also adjusted for duration of blood storage. [a]Progesterone concentrations are 0–15 days before next menstrual period. [b]Correlations between FSH and sex hormone concentrations are based on up to ten observations less than specified sample size.

**Table 3** Sex hormone serum concentrations in cases and control subjects

| Hormone (units) | Days until next menstrual period | Cases | | | Control subjects | | | P-value |
|---|---|---|---|---|---|---|---|---|
| | | $n$ | Mean | (95% CI) | $n$ | Mean | (95% CI) | |
| Oestradiol (pmol l⁻¹) | | | | | | | | |
| Early follicular | 22+ | 13 | 156 | (112–217) | 36 | 167 | (137–204) | 0.73 |
| Late follicular | 16–21 | 18 | 346 | (272–439) | 51 | 301 | (261–347) | 0.33 |
| Mid-cycle | 12–15 | 7 | 832 | (540–1281) | 24 | 475 | (376–600) | 0.04 |
| Early luteal | 3–11 | 12 | 394 | (282–441) | 35 | 398 | (350–454) | 0.94 |
| Late luteal | 0–2 | 11 | 256 | (181–362) | 33 | 223 | (183–273) | 0.51 |
| Whole cycle | Any | 61 | 316 | (276–362) | 179 | 283 | (262–307) | 0.17 |
| Progesterone (nmol l⁻¹) | | | | | | | | |
| Early follicular | 22+ | 14 | 4.14 | (2.98–5.77) | 39 | 4.96 | (4.07–6.04) | 0.37 |
| Late follicular | 16–21 | 18 | 2.59 | (1.99–3.36) | 51 | 2.37 | (2.03–2.77) | 0.59 |
| Mid-cycle | 12–15 | 7 | 6.24 | (3.14–12.4) | 24 | 8.04 | (5.55–11.6) | 0.54 |
| Early luteal | 3–11 | 12 | 28.5 | (20.7–39.2) | 35 | 31.2 | (25.8–37.6) | 0.64 |
| Late luteal | 0–2 | 11 | 12.9 | (8.28–20.2) | 33 | 12.5 | (9.64–16.1) | 0.89 |
| Testosterone (nmol l⁻¹) | Any | 62 | 1.25 | (1.12–1.40) | 182 | 1.20 | (1.13–1.28) | 0.57 |
| SHBG (nmol l⁻¹) | Any | 53 | 69.4 | (62.1–77.6) | 156 | 64.2 | (60.2–68.5) | 0.24 |

Values are geometric means. Concentrations of oestradiol and progesterone are adjusted for day of menstrual cycle in categories of 0–2, 3–5, 6–8, 9–11, 12–15, 16–18, 19–21, 22–24 and 25+ days before next menstrual period; concentrations of testosterone and SHBG are adjusted in categories of 0–2, 3–11, 12–15, 16–21 and 22+ days before next menstrual period. Concentrations of oestradiol, progesterone and testosterone are also adjusted for duration of blood storage.

family history of breast cancer, body mass index (BMI, kg $m^{-2}$), past use of oral contraceptives and the other hormone concentrations.

### RESULTS

Diagnosis of breast cancer was a mean of 8.0 years (range < 1–16 years) subsequent to blood collection. Breast cancer cases and control subjects were almost identical in age at interview (a matching criterion), age at menarche, length of menstrual cycle, weight and BMI (kg $m^{-2}$). Cases were on average 2 cm taller than control subjects. A lower proportion of cases than control subjects was parous, a higher proportion of cases reported a first-degree family history of breast cancer and past use of oral contraceptives, and a lower proportion of cases reported use of prescribed or over-the-counter medication at the time of interview. None of these differences was statistically significant (Table 1).

### Associations between hormones and other variables in control subjects

Oestradiol concentration increased significantly with increasing and testosterone concentration and decreased significantly with increasing FSH concentration (Table 2). Progesterone concentration measured 0–15 days before the next menstrual period decreased significantly with increasing FSH concentration. Testosterone concentration decreased significantly with increasing SHBG concentration and increased significantly with increasing BMI. SHBG concentration decreased significantly with increasing BMI.

The mean concentration of testosterone was 12% lower ($P$ = 0.06) and the mean concentration of SHBG was 14% lower ($P$ = 0.04) in women who had used oral contraceptives in the past. There were no statistically significant associations between hormone concentrations and parity (parous, nulliparous), first-degree family history of breast

Table 4 Odds ratios for breast cancer in relation to serum hormone concentrations

| Hormone | OR | (95% CI) | P-value |
|---|---|---|---|
| Oestradiol | 1.51 | (0.87–2.63) | 0.14 |
| Progesterone[a] | 0.76 | (0.37–1.53) | 0.44 |
| Testosterone | 1.22 | (0.61–2.43) | 0.57 |
| SHBG | 1.78 | (0.77–4.12) | 0.17 |

Odds ratios are estimated by conditional logistic regression for matched case–control sets and are based on a 1 unit increase in the natural log of hormone concentration. [a]Progesterone concentrations are 0–15 days before next menstrual period.

cancer, age at menarche, length of menstrual cycle, number of years since last use of oral contraceptives or use of prescribed or over-the-counter medication at the time of interview (results not shown).

### Hormone concentrations in cases and control subjects

Table 3 shows that, in comparison with the control subjects, the cases had a 12% higher mean oestradiol concentration ($P = 0.17$), 4% higher testosterone concentration ($P = 0.57$) and 8% higher SHBG concentration ($P = 0.24$) across the whole menstrual cycle. The reported results were adjusted for duration of blood storage and day of menstrual cycle, although the unadjusted results were similar, since these were matching criteria. Neither the exclusion of 29 breast cancer cases who were aged 50 years or older at diagnosis and their matched control subjects nor the exclusion of ten cases who donated blood less than 3 years before diagnosis and their matched control subjects appreciably altered the difference in mean oestradiol or testosterone concentrations between the cases and control subjects. However, after excluding the ten cases who donated blood less than 3 years before diagnosis and their matched control subjects, the mean concentration of SHBG was 16% higher in the cases than in the control subjects ($P = 0.04$).

The concentrations of oestradiol and progesterone were compared in the breast cancer cases and control subjects by five stages of the menstrual cycle. The mean oestradiol concentration was 75% higher in the cases than in the control subjects during the mid-cycle phase ($P = 0.04$), and between 7% lower and 15% higher in the cases at other stages of the cycle. The mean progesterone concentration was 9% lower in the cases during the early luteal phase ($P = 0.64$) and 3% higher in the cases during the late luteal phase ($P = 0.89$).

The odds ratios for breast cancer in relation to a unit increase in the log of the hormone concentrations of oestradiol, progesterone, testosterone and SHBG are presented in Table 4. None of the tests for linear trend was statistically significant. An odds ratio for the difference between the median concentration in the control subjects within the lowest third of the hormone distribution and the median concentration in the control subjects within the highest third of the distribution could be calculated for each hormone. The odds ratios for breast cancer in women with relatively high serum concentrations compared with women with lower concentrations of the hormones were: follicular phase oestradiol (16+ days before next menstrual period), OR = 1.71; luteal phase oestradiol (0–11 days before next menstrual period), OR = 1.67; progesterone (0–15 days before next menstrual period), OR = 0.58; testosterone, OR = 1.19; SHBG, OR = 1.58.

The odds ratios for oestradiol, progesterone and testosterone were not substantially altered by any adjustments. After adjusting SHBG concentration for past use of oral contraceptives, the odds ratio rose to 2.45 (95% CI 1.01–5.89, $P = 0.05$).

### DISCUSSION

These data are from a prospective study designed to investigate associations between serum concentrations of sex hormones in premenopausal women and risk of developing breast cancer. The blood samples in this study were collected a mean of 8.0 years before breast cancer diagnosis, and restriction of the analyses to cases who donated blood 3 or more years before diagnosis produced similar results to those quoted, so it is unlikely that the small differences in serum hormone concentrations are caused by the metabolic effects of early cancer. The results reliably represent hormone measurements of premenopausal women, since women with high serum concentrations of FSH were excluded. The negative correlation of oestradiol and progesterone concentrations with FSH concentration was observed at all ages and was not a result of decreasing oestradiol and progesterone and increasing FSH concentration as women approached the menopause.

Our data suggest that the mean oestradiol concentration over the complete menstrual cycle may be higher in women who develop breast cancer than in control subjects, but the small difference was not statistically significant. In the three other prospective studies published, Wysowski et al (1987) reported a 16% lower mean oestradiol concentration in breast cancer cases in samples taken throughout the cycle, Helzlsouer et al (1994) reported a 17% higher follicular phase oestradiol but a 29% lower luteal phase oestradiol concentration in the cases, and Rosenberg et al (1994) reported similar unadjusted mean oestradiol concentrations in the cases and control subjects, although the mean concentration was higher in the cases than the control subjects after adjusting for day of cycle by spline regression. The most noticeable finding from our study is the 75% higher mid-cycle serum oestradiol concentration in women who subsequently developed breast cancer compared with the control subjects, but this comparison was based on only seven cases, was not an a priori hypothesis and could simply be due to chance. Results for urinary oestrogen excretion (Key et al, 1996) in 35 of the 62 cases differed somewhat from those for serum oestradiol concentrations, but we believe serum concentrations to be a more reliable indicator of levels of oestradiol in the breast tissue. Unfortunately, the two measurements cannot be directly compared because the urine and blood samples from each woman were not collected on the same day of the menstrual cycle.

The concentration of SHBG is an important determinant of the proportion of oestradiol that is non-protein bound, which is thought to be the bioavailable fraction and has been hypothesized to be inversely related to breast cancer risk (Siiteri et al, 1981). Moore et al (1986) reported a 13% lower mean SHBG concentration in a subset of 12 premenopausal cases (which are also included in this study) than in the control subjects. However, Helzlsouer et al (1994) reported virtually identical concentrations of SHBG in cases and control subjects and we found a non-significantly higher mean SHBG concentration in the cases than in the control subjects. After adjusting for past use of oral contraceptives, our reported odds ratio for breast cancer in relation to SHBG concentration increased and was statistically significant. We report a 14% lower concentration of SHBG in women who had used oral

 © Cancer Research Campaign 1997

contraceptives in the past compared with those who had not, whereas Key et al (1989) found only a 4% lower concentration among 1243 premenopausal women within the Guernsey cohort who had previously used oral contraceptives compared with 616 women who had not. So, although our results seem contrary to the hypothesis for SHBG and breast cancer risk, it is still unclear how past use of oral contraceptives, SHBG concentration and risk of premenopausal breast cancer are truly associated.

Prospective epidemiological studies have provided only meagre evidence for an association between raised luteal phase concentrations of progesterone and breast cancer risk. Wysowski et al (1987) found 29% lower levels of progesterone in cases than in control subjects in samples taken throughout the cycle but did not report data for luteal phase concentrations. Helzlsouer et al (1994) reported a 94% higher median luteal phase concentration of progesterone in women who developed breast cancer than in control subjects, based on only nine cases. We found almost identical serum concentrations of progesterone in the cases and the control subjects during the luteal phase of the cycle.

Several case–control studies have reported an association between raised concentrations of testosterone and premenopausal breast cancer risk, but the only two prospective studies that have reported measurement of serum testosterone concentrations have not supported this hypothesis. Wysowski et al (1987) found no difference in mean testosterone concentrations and we report a non-significant 4% higher mean concentration of testosterone in the breast cancer cases than in the control subjects.

Our data include a relatively large number of premenopausal breast cancer cases in a prospective study of endogenous sex hormones and breast cancer. We found the mean oestradiol concentration to be 12% higher in the cases than in the control subjects. Although this small difference was not statistically significant, it could, nevertheless, be aetiologically important. It is clear that larger prospective studies are needed to clarify these results, perhaps using multiple blood samples collected at different phases of the menstrual cycle.

### ACKNOWLEDGEMENTS

We thank the following: Dr Richard Bulbrook and Mr John Hayward for the initiation, design and management for many years of the series of studies in Guernsey; the women of Guernsey who volunteered for this study; the general practitioners of Guernsey, Dr Bryan Gunton-Bunn, Dr David Jeffs, Miss Louise Davies and the staff at The Greffe for assistance in follow-up; Professor John Keenan and staff at the Biochemistry Laboratory, Radcliffe Infirmary, Oxford for technical assistance in measuring the FSH concentrations; Professor Valerie Beral for comments and advice. This study was funded by the Imperial Cancer Research Fund. DYW is funded by the Breast Cancer Research Trust.

### REFERENCES

Bernstein L, Yuan JM, Ross RK, Pike MC, Hanisch R, Lobo R, Stanczyk F, Gao Y-T and Henderson BE (1990) Serum hormone levels in pre-menopausal Chinese women in Shanghai and white women in Los Angeles: results from two breast cancer case–control studies. *Cancer Causes Control* **1**: 51–58

Berrino F, Muti P, Micheli A, Bolelli G, Krogh V, Sciajno R, Pisani P, Panico S and Secreto G (1996) Serum sex hormone levels after menopause and subsequent breast cancer. *J Natl Cancer Inst* **88**: 291–296

Dorgan JF, Longcope C, Stephenson HE, Falk RT, Miller R, Franz C, Kahle L, Campbell WS, Tangrea JA and Schatzkin A (1996) Relation of prediagnostic serum estrogen and androgen levels to breast cancer risk. *Cancer Epidemiol Biomarkers Prev* **5**: 533–539

Hammond GL, Langley MS and Robinson PA (1985) A liquid-phase immunoradiometric assay for human sex-hormone-binding-globulin. *J Steroid Biochem* **23**: 451–460

Helzlsouer KJ, Alberg AJ, Bush TL, Longcope C, Gordon GB and Comstock GW (1994) A prospective study of endogenous hormones and breast cancer. *Cancer Detect Prev* **18**: 79–85

Key TJA and Pike MC (1988) The role of oestrogens and progestagens in the epidemiology and prevention of breast cancer. *Eur J Cancer Clin Oncol* **24**: 29–43

Key TJA, Pike MC, Moore JW, Bulbrook RD, Clark GMG, Allen DS and Wang DY (1989) The relationships of SHBG with current and previous use of oral contraceptives and oestrogen replacement therapy. *Contraception* **39**: 179–186

Key TJA, Wang DY, Brown JB, Hermon C, Allen DS, Moore JW, Bulbrook RD, Fentiman IS and Pike MC (1996) A prospective study of urinary oestrogen excretion and breast cancer risk. *Br J Cancer* **73**: 1615–1619

Malarkey WB, Schroeder LL, Stevens VC, James AG and Lanese RR (1977) Twenty-four-hour preoperative endocrine profiles in women with benign and malignant breast disease. *Cancer Res* **37**: 4655–4659

Moore JW, Clark GMG, Hoare SA, Millis RR, Hayward JL, Quinlan MK, Wang DY and Bulbrook RD (1986) Binding of oestradiol to blood proteins and aetiology of breast cancer. *Int J Cancer* **38**: 625–630

Moore JW, Key TJA, Bulbrook RD, Clark GMG, Allen DS, Wang DY and Pike MC (1987) Sex hormone binding globulin and risk factors for breast cancer in a population of normal women who had never used exogenous sex hormones. *Br J Cancer* **56**: 661–666

Pike MC, Spicer DV, Dahmoush L and Press MF (1993) Estrogens, progestogens, normal breast cell proliferation, and breast cancer risk. *Epidemiol Rev* **15**: 17–35

Rosenberg CR, Pasternack BS, Shore RE, Koenig KL and Toniolo PG (1994) Premenopausal estradiol levels and the risk of breast cancer: a new method of controlling for day of the menstrual cycle. *Am J Epidemiol* **140**: 518–525

Secreto G, Recchione C, Fariselli G and Di Pietro S (1984) High testosterone and low progesterone circulating levels in premenopausal patients with hyperplasia and cancer of the breast. *Cancer Res* **44**: 841–844

Secreto G, Toniolo P, Pisani P, Recchione C, Cavalleri A, Fariselli G, Totis A, Di Pietro S and Berrino F (1989) Androgens and breast cancer in premenopausal women. *Cancer Res* **49**: 471–476

Siiteri PK, Hammond GL and Nisker JA (1981) Increased availability of serum estrogens in breast cancer: a new hypothesis. In *Hormones and Breast Cancer*, Pike MC, Siiteri PK and Welsch CW (eds), pp. 87–106. Cold Spring Harbor Laboratory Press: New York

Toniolo PG, Levitz M, Zeleniuch-Jacquotte A, Banerjee S, Koenig KL, Shore RE, Strax P and Pasternack BS (1995) A prospective study of endogenous estrogens and breast cancer in postmenopausal women. *J Natl Cancer Inst* **87**: 190–197

Wysowski DK, Comstock GW, Helsing KJ and Lau HL (1987) Sex hormone levels in serum in relation to the development of breast cancer. *Am J Epidemiol* **125**: 791–799

*© Cancer Research Campaign 1997*